# Development and external validation of a novel nomogram to predict prostate cancer in biopsy‐naïve patients with PSA <10 ng/ml and PI‐RADS v2.1 = 3 lesions

**DOI:** 10.1002/cam4.5100

**Published:** 2022-08-03

**Authors:** Can Hu, Jiale Sun, Zhenyu Xu, Zhiyu Zhang, Qi Zhou, Jiangnan Xu, Hao Chen, Chao Wang, Jun Ouyang

**Affiliations:** ^1^ Department of Urology The First Affiliated Hospital of Soochow University Suzhou Jiangsu China; ^2^ Department of Urology The Affiliated Hospital of Nanjing University of Traditional Chinese Medicine Kunshan China

**Keywords:** csPCa, equivocal lesions, nomogram, PI‐RADS v2.1

## Abstract

**Objective:**

To develop and externally validate a novel nomogram in biopsy‐naïve patients with prostate‐specific antigen (PSA) <10 ng/ml and PI‐RADS v2.1 = 3 lesions.

**Methods:**

We retrospectively collected 307 men that underwent initial biopsy from October 2015 to January 2022 in Cohort 1 (The First Affiliated Hospital of Soochow University). External cohort (Cohort 2, Kunshan Hospital) included 109 men that met our criteria from July 2016 to June 2021. By Slicer‐3D Software, the volume of all lesions was divided into two subgroups (PI‐RADS v2.1 = 3a and 3b). Logistic regression analysis was performed to screen for variables and construct nomogram by analyzing clinical data from Cohort 1. Receiver operating characteristics curve analysis, calibration plot and decision curve analysis (DCA) were plotted to validate the nomogram in external cohort.

**Results:**

A total of 70 (22.8%) patients was diagnosed with prostate cancer in Institution 1. Among them, 34 (11.1%) had clinically significant prostate cancer (csPCa). Age, prostate‐specific antigen density, digital rectal examination, PI‐RADS v2.1 = 3 subgroups (3a and 3b) and apparent diffusion coefficient (ADC, <750 mm^2^/s) were predictive factors for prostate cancer (PCa) and csPCa. High area under the curve of the nomogram was found in Cohort 1 and Cohort 2 for PCa (0.857 vs. 0.850) and for csPCa (0.896 vs. 0.893). Calibration curves showed excellent agreement between the predicted probability and actual risk for the models in internal and external validation. The DCA demonstrated net benefit of our nomogram.

**Conclusion:**

Until now, this is the first nomogram that predicts PCa and csPCa in biopsy‐naïve patients with PSA <10 ng/ml and PI‐RADS v2.1 = 3 lesions. Furthermore, PI‐RADS v2.1 = 3 subgroups were considered to be an independent risk factor in our model. Our nomogram may assist urologists in biopsy decision making for these so‐called “double gray zone” patients.

## Graphical abstract

To our knowledge, this is the first study that applied PI‐RADS 3 subgroup into nomogram. Overall, the nomogram is a promising tool for predicting prostate cancer and clinically significant prostate cancer in biopsy‐naïve patients with PSA <10 ng/ml and PI‐RADS v2.1 = 3 lesions due to the good calibration and net benefit. Our model may assist urologists in biopsy decision making for the “double gray zone” patients.

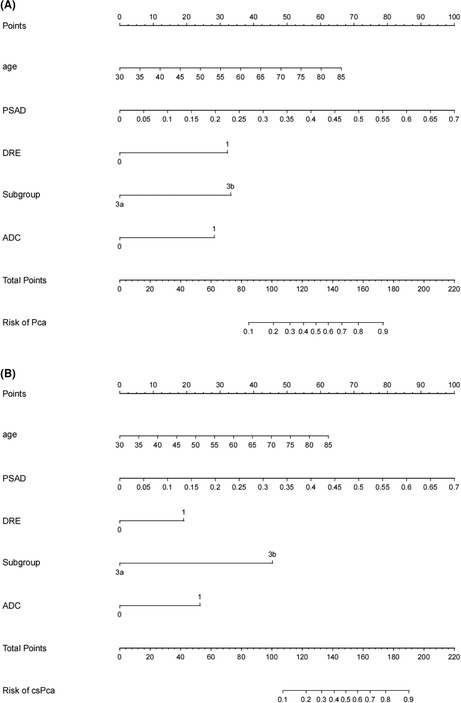

## 1 INTRODUCTION

Prostate cancer (PCa) is the most common tumor of male urogenital system. Up to now, the morbidity of PCa ranked second of all malignancies in men worldwide.^1^ Although the morbidity and mortality of PCa in Asia are relatively lower than those in Europe and the United States, the trend is obviously rising in recent years with the wide application of screening test such as serum prostate‐specific antigen (PSA) and multiparametric magnetic resonance imaging (mpMRI).^2^ mpMRI was identified as the superior method for the detection and assessment of PCa and clinically significant prostate cancer (csPCa).^3^ Thus, based on mpMRI, the Prostate Imaging Reporting and Data System has been designed in 2012 and updated to version 2.1 (PI‐RADS v2.1) in 2019.^4^ PI‐RADS standardizes the assessment of mpMRI and reports conclusive results for reference of clinicians to select patients for biopsies and management, which utilizes a 5‐point scale to represent the risk of csPCa. Among the five scales, PI‐RADS v2.1 category 3 (PI‐RADS 3) lesions are generally considered to be equivocal or ambiguous.^5^, ^6^ Therefore, there is no agreement on the clinical management (biopsy or clinical surveillance) of patients receiving PI‐RADS 3.

Although the European Association of Urology 2021 guidelines recommended that biopsy should be performed when magnetic resonance imaging (MRI) is positive (PI‐RADS ≥3), the diagnostic accuracy of the procedure combined PSA with mpMRI remains to be discussed especially in patients with PSA <10 ng/ml and PI‐RADS 3 lesions.^7^, ^8^, ^9^ The current studies have shown an obvious difference on the detection rate of PCa in biopsied PI‐RADS 3 lesions varying from 5% to 46%^6^, ^9^, ^10^ in spite of different definitions of csPCa and methods of biopsy. Thus, characterizing these equivocal lesions appears to be particularly important. Prostate cancer is a visual three‐dimensional lesion and its characteristics are better represented by volume than tumor diameter only.^11^, ^12^ Besides, the strong correlation between lesion volume (LV) (measured by MRI) and tumor volume (measured by RP specimen) was confirmed in earlier studies.^11^, ^13^, ^14^ In PI‐RADS v2.0, LV ≥0.5 ml was incorporate into the definition of csPCa.^15^ After considering the location and background tissue of the lesion, intermediate or high‐grade cancers with volumes ≥0.5 ml could be detected by mpMRI. Hence in addition to pathology/histology as Gleason score ≥7, csPCa is also defined on volume ≥0.5 ml and/or extra prostatic extension. In conclusion, the cut‐off value of 0.5 ml has the ability to identify the equivocal PI‐RADS 3 lesions. In our study, with a cut‐off of 0.5 ml, the PI‐RADS 3 lesions was assigned into two subgroups: 3a (indolent or low‐risk lesions with volume <0.5 ml) and 3b (significant or high‐risk lesions with volume ≥0.5 ml).

Therefore, the objective of the study was to develop and externally validate a nomogram combined with PI‐RADS 3 subgroups and improve the detection of PCa or csPCa in men with PSA <10 ng/ml at first biopsy.

## 2 MATERIALS AND METHODS

### 2.1 Patients recruitment

With the approval of our institutional review board, we retrospectively evaluated the database of all biopsy‐naive patients to extract clinical records. A total of 366 men with PSA <10 ng/ml and PI‐RADS v2.1 = 3 lesions were enrolled in our study. All the patients underwent ultrasound guided transrectal or transperineal biopsies including 12 + *x* (8 cores in peripheral zone [PZ] and 4 cores in transitional zone [TZ]; *x* represents cores obtained from suspicious or positive areas) cores which performed by two experienced urologists. csPCa and clinically insignificant prostate cancer (cisPCa) were defined as International Society of Urogenital Pathology grade group ≥2 and grade group = 1, respectively. The exclusion criteria were listed as follows: (a) Any possible situations that influenced PSA such as prior medication for benign prostatic hyperplasia or indwelling catheters. (b) poor image quality of mpMRI. (c) repeated biopsy. (d) the edge of lesions could not be defined. After conducting the exclusion criteria, 307 men were identified to constitute our study cohort.

### 2.2 MRI protocol and LV calculation

mpMRI was done using 3 T MRI equipment (General Electric) and three main scan sequences were included: axial T2 weighted image (T2WI), Diffusion weighted imaging (DWI), and dynamic contrast enhancement images. As was recommended in PI‐RADS v2.1, apparent diffusion coefficient (ADC) value was calculated by the highest *b*‐value ≤1000 s/mm^2^ to avoid diffusion kurtosis effect.^16^ The level of diagnosis was almost the same in the two institutions. Blind to clinical data of all patients, all mpMRI scanning images were assessed by two radiologists with more than 5 years experience in prostate imaging. The index lesion was screened out and scored with the provisions of PI‐RADS v2.1. Mean ADC value of the index lesion was then calculated by drawing regions of interest. Slicer‐3D Software (v.4.10.0; http://slicer.org/) was applied to calculate the volume from axial T2WI. By setting the critical value of the image density parameter, two urologists used sphere brush function to distinguish the region of interest (ROI) from other areas (Supplementary S1). A semi‐automatic three‐dimensional segmentation image of the index lesion was then acquired and the software calculated the LV giving a value in milliliters (ml) automatically.^17^

### 2.3 Statistical analysis

Non‐normal distribution continuous variables were expressed as medians (interquartile range). Differences among groups were compared with using the Mann–Whitney test. Categorical variables were expressed as numbers (rates) and compared using chi square test. Univariate and multivariate binary logistic regression were applied to explore independent risk factors of PCa and csPCa, by which the nomogram was established. Receiver operator characteristic (ROC) curves were plotted by MedCalc and the area under the curves (AUC) was acquired to display the good predictive capability of the nomogram. Internal validation of the nomogram was conducted using 1000 bootstrap resamples (using Harrell's C‐index to quantify the discrimination performance). In external validation, calibration and clinical usefulness of the nomogram were assessed using the calibration plot and decision curve analysis (DCA). SPSS v.23.0 were used to conduct clinical baseline data analysis. The nomogram, calibration plot, and DCA curve were plotted by R software v.4.1.1. Two‐sided *p* < 0.05 was selected to indicate statistical significance.

## 3 RESULTS

### 3.1 Clinical characteristics and nomogram construction

**FIGURE 1:**
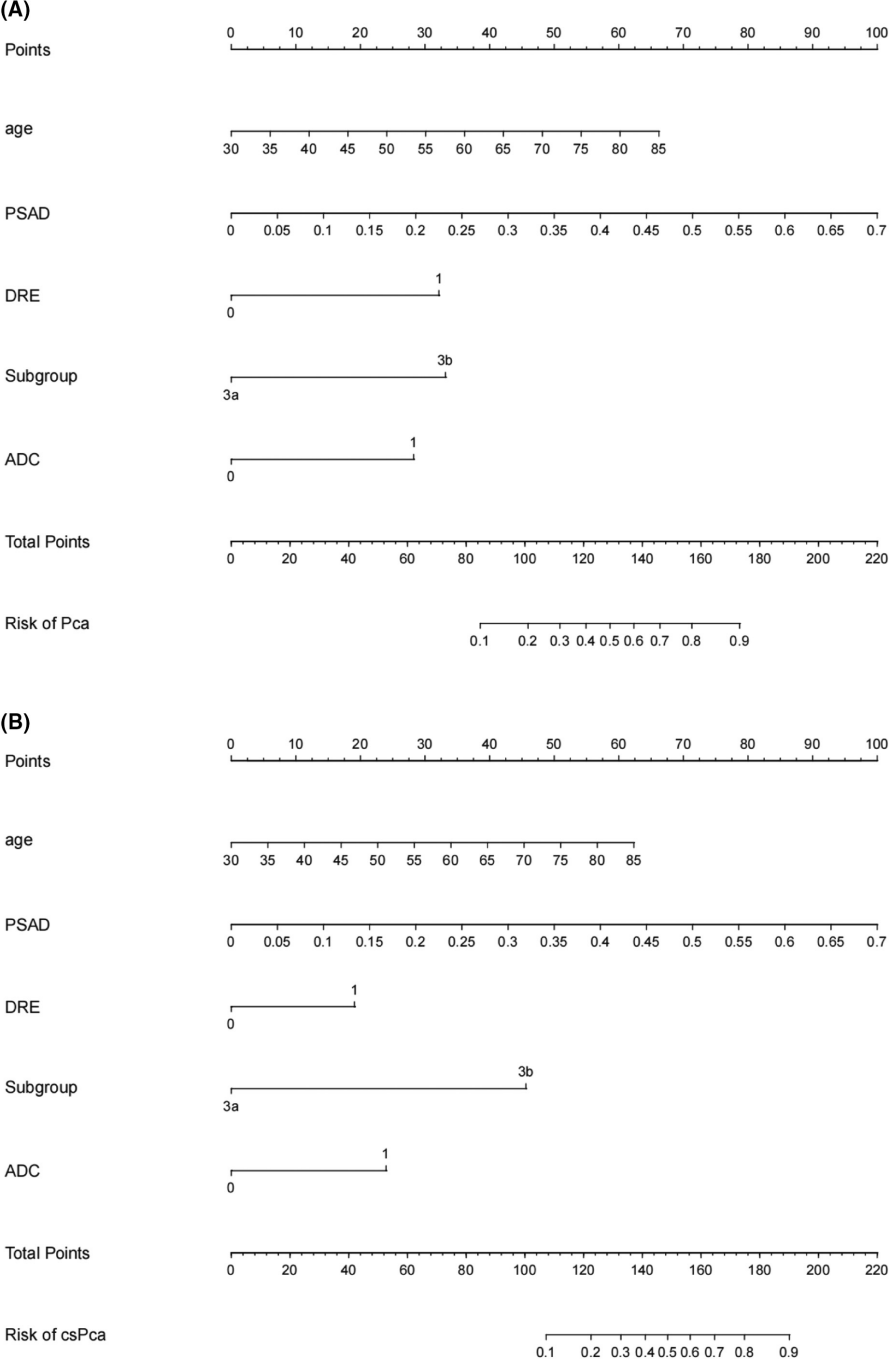
Nomogram predicting prostate cancer (A). Nomogram predicting clinically significant prostate cancer (B). Instructions: 1. Locate the five variables of patients on corresponding axis separately. 2. Then draw a vertical line of each variable upward to the “points” axis to determine how many points toward the probability of cancer. 3. Sum the points for each of the predictors and locate it on the “total points” axis. 4. Draw a line straight up to find the probability of PCa or csPCa. csPCa, clinically significant prostate cancer; PCa, prostate cancer.

All data of patients including demographic, clinical and imaging features in the two institutions were presented in Tables 1 and 2. Positive ADC value was considered as <750 mm^2^/s.^15^ Of the 307 men in Institution 1, 70 patients were diagnosed with PCa (22.8%) while 34 were confirmed as csPCa (11.1%). Compare to those without PCa, older age, lower free prostate‐specific antigen (fPSA), less percentage of free prostate‐specific antigen (%fPSA), smaller prostate volume, higher prostate‐specific antigen density (PSAD), more positive digital rectal examination (DRE) and ADC, larger LV and greater odds of PI‐RADS 3b were observed in patients with PCa or csPCa. Similar results were obtained in Institution 2 in which 109 patients were enrolled. The proportions of PCa and csPCa in Institution 2 were 22.9% and 13.7%, respectively. There was no significant difference in the ratio of PCa and csPCa between the two institutions. Independent risk factors of PCa and csPCa were explored by the analysis of univariable and multivariable binary logistic regression (Tables 3 and 4). Finally, age, PSAD, DRE, PI‐RADS 3 subgroups and ADC were screened out to establish the nomogram (Figure 1A,B).

**TABLE 1:** Comparison of variables between benign and PCa in the two centers

Variables	FAHSU^a^			KS^b^		
	Benign (*n* = 237)	PCa (*n* = 70)	*p* value	Benign (*n* = 84)	PCa (*n* = 25)	*p* value
Age, y (M, IQR)	65 (59, 72)	69 (61, 75)	**0.001**	68 (61, 68)	70 (68, 75)	0.239
tPSA, ng/ml (M, IQR)	7.08 (5.39, 9.08)	6.88 (5.42, 8.64)	0.528	7.78 (6.01, 9.53)	7.17 (6.55, 9.39)	0.986
fPSA, ng/ml (M, IQR)	1.08 (0.68, 1.46)	0.96 (0.59, 1.16)	**0.015**	1.33 (0.98, 1.91)	0.97 (0.69, 1.44)	**0.014**
%fPSA (M, IQR)	0.16 (0.12, 0.20)	0.12 (0.09, 0.16)	**0.002**	0.18 (0.14, 0.23)	0.14 (0.09, 0.19)	**0.002**
PV, ml (M, IQR)	45 (36, 57)	35 (25, 49)	**<0.001**	48 (35, 69)	38 (24, 51)	**0.008**
PSAD, ng/ml/g (M, IQR)	0.15 (0.10, 0.20)	0.18 (0.14, 0.25)	**<0.001**	0.15 (0.10, 0.22)	0.21 (0.15, 0.36)	**0.014**
NLR (M, IQR)	0.57 (0.39, 0.97)	0.57 (0.44, 0.74)	0.719	0.77 (0.55, 1.85)	0.51 (0.43, 0.65)	**<0.001**
PLR (M, IQR)	115 (70, 147)	112 (85, 142)	0.363	115 (92, 156)	113 (82, 145)	0.375
LMR (M, IQR)	3.8 (3.0, 5.1)	4.0 (3.1, 4.8)	0.704	4.2 (3.2, 5.4)	4.3 (3.5, 4.8)	0.746
Mesh volume, ml (M, IQR)	0.35 (0.23, 0.52)	0.61 (0.31, 0.81)	**<0.001**	0.36 (0.21, 0.49)	0.60 (0.29, 0.74)	**0.006**
DRE, *n*			**0.001**			**0.003**
Normal	170 (71.8%)	26 (37.1%)		58 (69.0%)	9 (36.0%)	
Suspicious	67 (28.2%)	44 (62.9%)		26 (31.0%)	16 (64.0%)	
Location, *n*			0.109			0.249
TZ	55 (23.1%)	10 (14.3%)		23 (27.4%)	4 (14.3%)	
PZ	182 (76.9%)	60 (85.7%)		61 (72.6%)	21 (85.7%)	
PIRADSv2.1 = 3 subgroup, *n*			**<0.001**			**<0.001**
3a	181 (76.4%)	27 (38.6%)		65 (77.4%)	10 (40.0%)	
3b	56 (23.6%)	43 (61.4%)		19 (22.6%)	15 (60.0%)	
ADC, mm^2^/s			**<0.001**			**<0.001**
≥750	211 (88.6%)	45 (64.3%)		73 (86.9%)	11 (44.0%)	
<750	26 (11.4%)	25 (35.7%)		11 (13.1%)	14 (66.0%)	

*Note*: Boldface indicates statistically significant difference.

Abbreviations: %fPSA, percentage of free prostate‐specific antigen; ADC, apparent diffusion coefficient; DRE, digital rectal examination; LMR, lymphocyte‐to‐monocyte ratio; NLR, neutrophil‐to‐lymphocyte ratio; PCa, prostate cancer; PIRADSv2.1, prostate imaging reporting and data system version 2.1; PLR, platelet‐to‐lymphocyte ratio; PSAD, prostate‐specific antigen density; PV, prostate volume; PZ, peripheral zone; TZ, transitional zone.

^a^ FAHSU: The First Affiliated Hospital of Soochow University.

^b^ KS: Kunshan Chinese Medicine Hospital Affiliated to Nanjing University of Chinese Medicine (External validation cohort).

**TABLE 2:** Comparison of variables between benign + cisPCa and csPCa in the two centers

Variables	FAHSU^a^			KS^b^		
	Benign+ cisPCa (*n* = 271)	csPCa (*n* = 34)	*p* value	Benign+ cisPCa (*n* = 94)	csPCa (*n* = 15)	*p* value
Age, y (M, IQR)	65 (59, 72)	69 (64, 75)	**0.001**	69 (65, 73)	70 (66, 75)	0.239
tPSA, ng/ml (M, IQR)	7.04 (5.29, 9.05)	7.80 (5.90, 9.00)	0.528	7.78 (6.09, 9.50)	7.10 (6.50, 9.22)	0.986
fPSA, ng/ml (M, IQR)	1.05 (0.67, 1.44)	0.98 (0.60, 1.14)	0.015	1.33 (0.98, 1.76)	0.87 (0.67, 1.44)	**0.014**
%fPSA (M, IQR)	0.15 (0.11, 0.20)	0.12 (0.09, 0.14)	**0.002**	0.18 (0.13, 0.22)	0.13 (0.07, 0.21)	**0.002**
PV, ml (M, IQR)	43 (34, 56)	35 (24, 49)	**<0.001**	47 (33, 66)	37 (23, 45)	**0.008**
PSAD, ng/ml/g (M, IQR)	0.15 (0.11, 0.21)	0.19 (0.15, 0.27)	**<0.001**	0.16 (0.11, 0.23)	0.22 (0.14, 0.39)	**0.014**
NLR (M, IQR)	0.58 (0.39, 0.90)	0.51 (0.40, 0.68)	0.719	0.72 (0.52, 1.84)	0.49 (0.42, 0.52)	**<0.001**
PLR (M, IQR)	115 (90, 147)	108 (80, 131)	0.363	112 (91, 154)	122 (78, 147)	0.375
LMR (M, IQR)	3.9 (3.0, 5.0)	4.0 (3.0, 4.8)	0.704	4.1 (3.2, 5.3)	4.4 (3.2, 4.9)	0.746
Mesh volume, ml (M, IQR)	0.36 (0.23, 0.59)	0.67 (0.59, 0.84)	**<0.001**	0.35 (0.21, 0.49)	0.65 (0.59, 0.88)	**0.006**
DRE, *n*			**0.001**			**0.003**
Normal	185 (68.3%)	13 (38.2%)		63 (67.0%)	4 (26.7%)	
Suspicious	86 (31.7%)	21 (61.8%)		31 (33.0%)	11 (73.3%)	
Location, *n*			0.109			0.249
TZ	61 (22.5%)	4 (11.8%)		25 (26.6%)	2 (13.3%)	
PZ	210 (77.5%)	30 (88.2%)		69 (73.4%)	13 (86.7%)	
PIRADSv2.1 = 3 subgroup, *n*			**<0.001**			**<0.001**
3a	202 (74.8%)	7 (20.6%)		71 (75.5%)	4 (26.7%)	
3b	69 (26.2%)	27 (79.4%)		23 (24.5%)	11 (73.3%)	
ADC, mm^2^/s			**<0.001**			**<0.001**
≥750	236 (87.1%)	20 (58.8%)		79 (84.0%)	5 (33.3%)	
<750	35 (12.9%)	14 (41.2%)		15 (16.0%)	10 (66.7%)	

*Note*: Boldface indicates statistically significant difference.

Abbreviations: %fPSA, percentage of free prostate‐specific antigen; ADC, apparent diffusion coefficient; csPCa, clinically significant prostate cancer; DRE, digital rectal examination; LMR, lymphocyte‐to‐monocyte ratio; NLR, neutrophil‐to‐lymphocyte ratio; PCa, prostate cancer; PIRADSv2.1, prostate imaging reporting and data system version 2.1; PLR, platelet‐to‐lymphocyte ratio; PSAD, prostate‐specific antigen density; PV, prostate volume; PZ, peripheral zone; TZ, transitional zone.

^a^ FAHSU: The First Affiliated Hospital of Soochow University.

^b^ KS: Kunshan Chinese Medicine Hospital Affiliated to Nanjing University of Chinese Medicine (External validation cohort).

**TABLE 3:** Univariable and multivariable binary logistic regression analysis testing variables as independent risk factors of PCa

Variables	Univariable analysis		Multivariable analysis	
	Odds ratio (95% CI)	*p* value	Odds ratio (95% CI)	*p* value
Age	1.053 (1.019–1.088)	**0.002**	1.080 (1.034–1.128)	**<0.001**
fPSA	0.576 (0.354–0.940)	**0.027**	0.075 (0.224–2.514)	0.641
%fPSA	0.009 (0.000–0.431)	**0.017**	0.035 (0.000–145.460)	0.430
Volume	0.976 (0.961–0.993)	**0.004**	0.993 (0.967–1.020)	0.622
PSAD	70.207 (4.759–1035.701)	**0.002**	260.261 (1.654–40, 951.975)	**0.031**
DRE	4.478 (2.551–7.860)	**<0.001**	4.695 (2.383–9.251)	**<0.001**
Subgroup	5.270 (2.986–9.300)	**<0.001**	4.794 (2.420–9.496)	**<0.001**
ADC	4.509 (2.386–8.519)	**<0.001**	3.784 (1.728–8.285)	**0.001**

Abbreviations: %fPSA, percentage of free prostate‐specific antigen; ADC, apparent diffusion coefficient; DRE, digital rectal examination; fPSA, free prostate‐specific antigen; PCa, prostate cancer; PSAD, prostate‐specific antigen density.

**TABLE 4:** Univariable and multivariable binary logistic regression analysis testing variables as independent risk factors of csPCa

Variables	Univariable analysis		Multivariable analysis	
	Odds ratio (95% CI)	*p* value	Odds ratio (95% CI)	*p* value
Age	1.058 (1.013–1.106)	**0.012**	1.083 (1.018–1.153)	**0.012**
fPSA	0.694 (0.374–1.288)	0.247	2.571 (0.377–17.539)	0.335
%fPSA	0.002 (0.000–0.554)	**0.031**	0.000 (0.000–3.160)	0.070
Volume	0.984 (0.965–1.005)	0.130	1.003 (0.973–1.033)	0.866
PSAD	102.705 (5.332–1978.204)	**0.002**	789.192 (2.365–263, 380.984)	**0.024**
DRE	3.396 (1.625–7.095)	**0.001**	3.118 (1.260–7.718)	**0.014**
Subgroup	10.974 (4.578–26.303)	**<0.001**	11.433 (4.160–31.426)	**<0.001**
ADC	4.465 (2.076–9.603)	**<0.001**	3.537 (1.363–9.181)	**0.009**

Abbreviations: %fPSA, percentage of free prostate‐specific antigen; ADC, apparent diffusion coefficient; csPCa, clinically significant prostate cancer; DRE, digital rectal examination; fPSA, free prostate‐specific antigen; PSAD, prostate‐specific antigen density.

### 3.2 Nomogram evaluation

**FIGURE 2:**
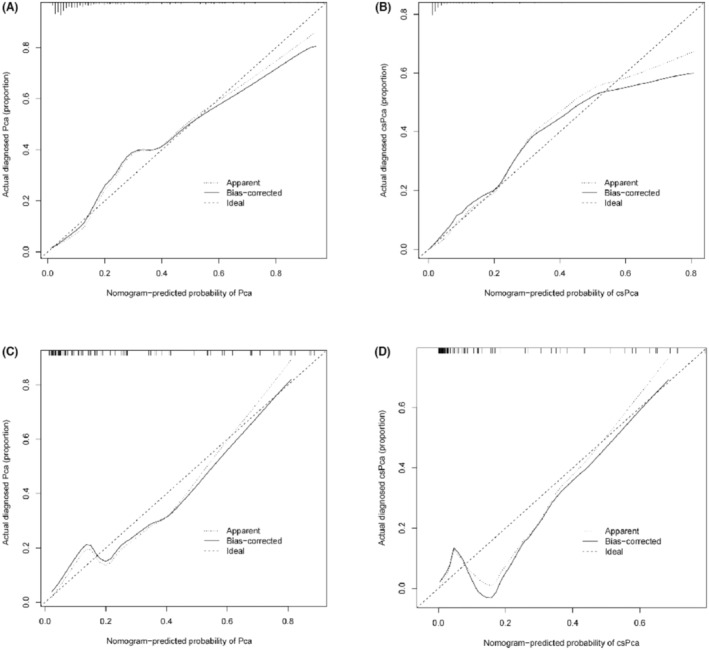
Calibration curve of the nomogram. The institution 1 for PCa (A) and csPCa (B); The external validation group for PCa (C) and csPCa (D). csPCa, clinically significant prostate cancer; PCa, prostate cancer.

**FIGURE 3:**
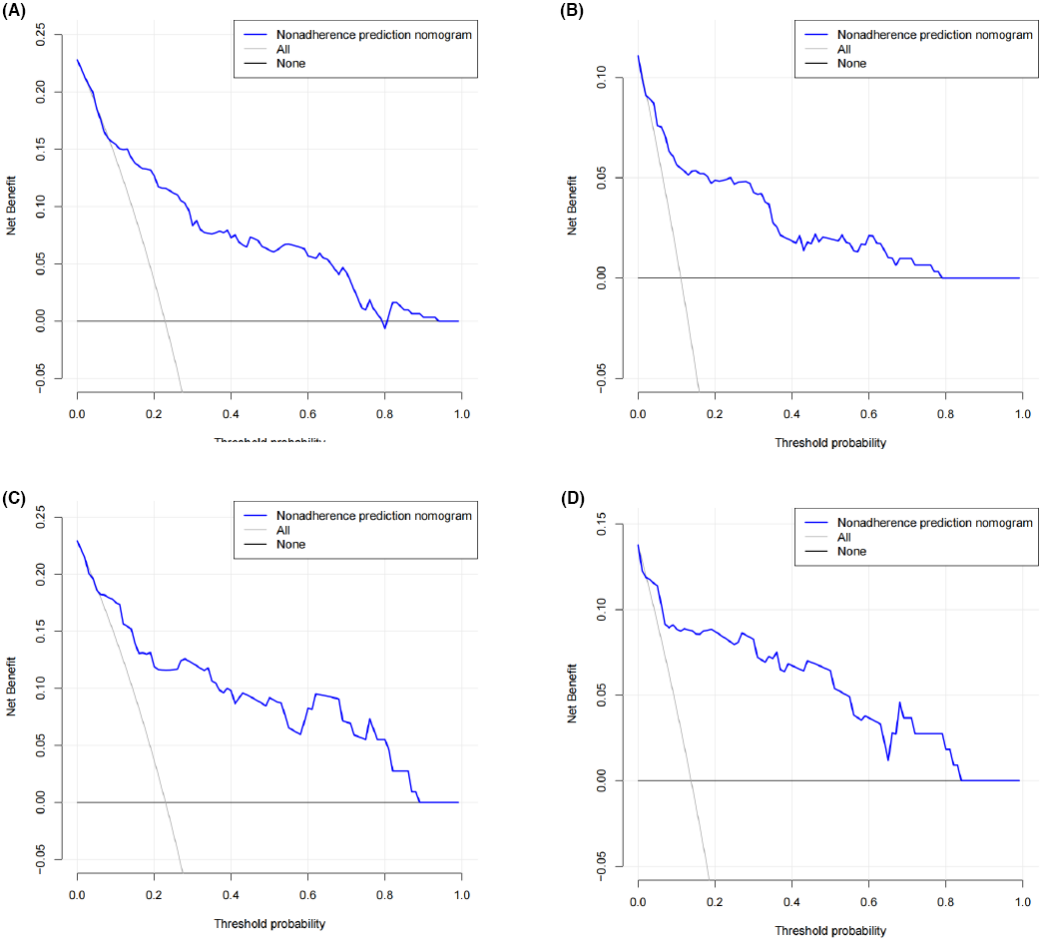
DCA of the nomogram. The institution 1 for PCa (A) and csPCa (B); The external validation group for PCa (C) and csPCa (D). csPCa, clinically significant prostate cancer; DCA, decision curve analyses; PCa, prostate cancer.

With the method of using 1000 bootstrap resamples, our model displayed good discrimination with a C‐index of 0.856 for PCa and 0.887 for csPCa. High C‐index value of 0.845 and 0.877 could still be observed in the internal validation. In the institution 1 (Figure 2A,B) and external validation group (Figure 2C,D), good concordance was seen in calibration curves between the predicted probability and the actual probability of the models. Besides, the nomogram might underestimate the risk when the threshold was approximately between 18% and 78% for both PCa and csPCa in external validation group. The DCA of institution 1 was presented in Figure 3A,B. The DCA of external validation group was presented in Figure 3C,D. In conclusion, both of the two models could achieve better net benefit than the intervention‐all‐patients scheme or the intervention‐none scheme even when the threshold was <10% or >80% in external validation group.

**FIGURE 4:**
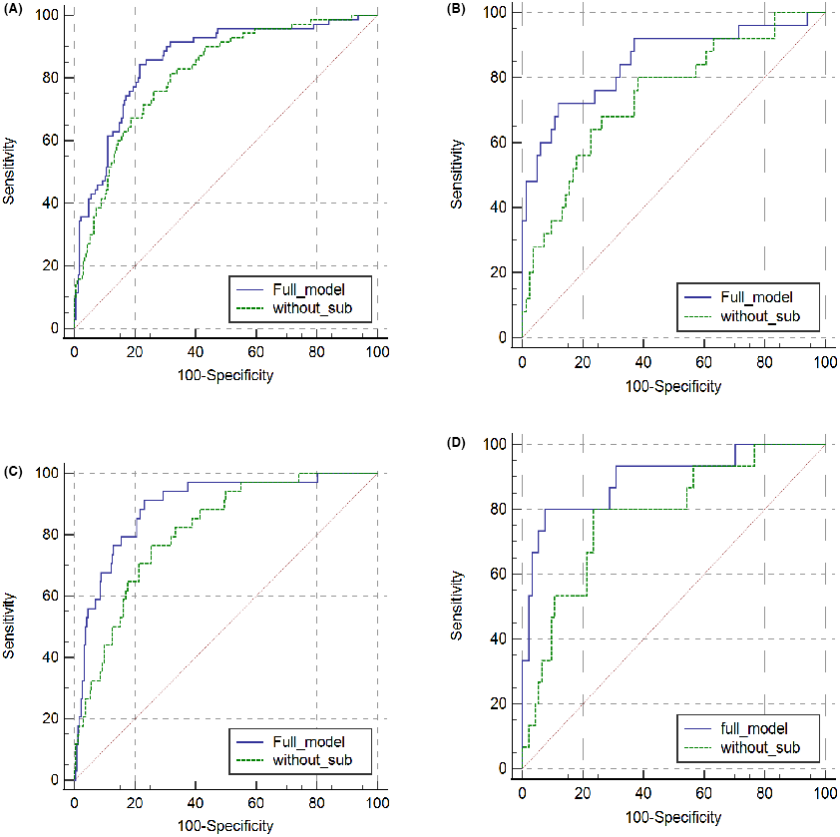
ROC curve comparing full model with model without PI‐RADS 3 subgroups for PCa (A) and for csPCa (B) in institution 1 and for PCa (C) and for csPCa (D) in the external cohort. csPCa, clinically significant prostate cancer; PCa, prostate cancer; ROC, receiver operating characteristic.

The ROC curve analysis illustrated that the application of PI‐RADS 3 subgroups increased the AUC of the model for PCa and especially for csPCa both in institution 1 and external cohort (Figure 4A–D; Tables 5 and 6). AUC for predicting PCa and csPCa were 0.857 (95% CI 0.768–0.911, *p* < 0.001) and 0.896 (95% CI 0.823–0.947, *p* < 0.001) in external cohort, respectively.

**TABLE 5:** ROC curve comparing full model with model without PI‐RADS 3 subgroups for PCa

	Full model				Without PI‐RADS 3 subgroups			
	AUC	SEN (%)	SPE (%)	*p*	AUC	SEN	SPE	*p*
Cohort 1	0.857	84.3	78.5	<0.001	0.814	81.4	68.4	<0.001
External validation group	0.850	72.0	88.1	<0.001	0.745	80.0	61.9	<0.001

Abbreviations: AUC, area under the curve; PCa, prostate cancer.

**TABLE 6:** ROC curve comparing full model with model without PI‐RADS 3 subgroups for csPCa

	Full model				Without PI‐RADS 3 subgroups			
	AUC	SEN (%)	SPE (%)	*p*	AUC	SEN	SPE	*p*
Cohort 1	0.893	91.2	76.9	<0.001	0.813	76.5	74.7	<0.001
External validation group	0.896	80.0	92.6	<0.001	0.784	80.0	76.6	<0.001

Abbreviations: AUC, area under the curve; csPCa, clinically significant prostate cancer.

Based on our risk model, in external cohort, there was a considerable reduction in unnecessary biopsies at a relatively low cost of missing PCa and csPCa (Tables 7 and 8). the positive predictive value (PPV) and negative predictive value (NPV) for PCa were 62.1% and 91.3% when the threshold of the nomogram was 0.34 (the highest Youden index of the model). The PPV and NPV for csPCa were 60.0% and 96.6% when the criterion was 0.26.

**TABLE 7:** The results of different probability cut‐offs calculated by our multivariable risk model for PCa in external validation group

Probability cut‐off (%)	Biopsies performed, *n*	Biopsies not performed, *n*	Unnecessary biopsies avoided, *n* (%)	PCa missed, *n* (%)
5	73	36	34 (31.2)	2 (1.8)
10	58	51	49 (50.0)	2 (1.8)
15	47	62	57 (52.3)	5 (4.6)
20	38	71	64 (58.7)	7 (6.4)
25	34	75	68 (62.4)	7 (6.4)
30	29	80	73 (70.0)	7 (6.4)
34	29	80	73 (67.0)	7 (6.4)

Abbreviation: PCa, prostate cancer.

**TABLE 8:** The results of different probability cut‐offs calculated by our multivariable risk model for csPCa in external validation group

Probability cut‐off (%)	Biopsies performed, *n*	Biopsies not performed, *n*	Unnecessary biopsies avoided, *n* (%)	csPCa missed, *n* (%)
5	43	66	65 (59.6)	1 (0.9)
10	33	76	73 (67.0)	3 (2.7)
15	27	82	79 (72.5)	3 (2.7)
20	22	87	84 (77.0)	3 (2.7)
26	20	89	86 (79.0)	3 (2.7)
30	19	90	87 (80.0)	3 (2.7)

Abbreviation: csPCa, clinically significant prostate cancer.

## 4 DISCUSSION

As the most commonly used clinical screening indicator, PSA <10 ng/ml was considered to be a gray zone for biopsy at present.^18^ Generally, when eliminating any possible situations that influenced PSA, we do not attach much importance to PI‐RADS scores and take a positive attitude toward biopsy if PSA >10 ng/ml.^19^ Recently, PI‐RADS 3 was identified as another gray zone for PCa due to the different detection rate.^10^, ^20^ To our knowledge, there were few studies reporting on the outcomes of prostate biopsy in the so‐called “double gray zone” cohort.^21^, ^22^ Thus, the management of this kind of patients remains to be controversial in clinical practice. In this study, therefore, we developed and validated a nomogram to predict the risk of PCa and csPCa in the “double gray zone” cohort, allowing appropriate management recommendations.

Age and DRE are commonly used to predict PCa and csPCa in many previous studies. Sheridan et al.^23^ constructed risk stratification of PI‐RADS 3 lesions and identified age, DRE as risk factors for csPCa. Meanwhile, Radtke et al.^20^ built a nomogram (AUC = 0.84) containing the two parameters by evaluating a cohort of 1159 men. Besides, PSAD has been proven to contribute to predict biopsy outcome.^24^, ^25^ In the present study, when setting 0.15 as a cut‐off, the sensitivity, specificity, PPV and NPV for csPCa were 0.82, 0.46, 0.16, 0.96, respectively. However, there were varying degrees of improvement in the four indexes (the sensitivity, specificity, PPV and NPV for csPCa were 0.91, 0.77, 0.32, 0.98, respectively). Given the relatively low detection rate in our “double gray zone” cohort, the low PPV was acceptable. Furthermore, Kund et al.^26^ reported that PSAD had a strong correlation with prostate cancer aggressiveness, such as tumor volume. This conclusion was demonstrated by Rico et al.^27^ who divided patients into four groups according to PSAD cut‐off and PI‐RADS 3 subgroups. Collectively, the three indicators above were convenient clinical parameters in predicting biopsy outcome while PSAD had a better performance in suggesting aggressive prostate cancers by means of combining tumor volume.

To our knowledge, this is the first study that applied PI‐RADS 3 subgroup into nomogram. The full nomogram outperforms the model without PI‐RADS 3 subgroup in diagnosing PCa (AUC = 0.857 vs. 0.814 in the model‐building group; AUC = 0.850 vs. 0.745 in the external validation group) and csPCa (AUC = 0.893 vs. 0.813 in the model‐building group; AUC = 0.896 vs. 0.784 in the external validation group). In the beginning, Stamey et al.^28^reported that a threshold of 0.5 ml on radical prostatectomy specimens was less likely to become csPCa. Whereafter, Epstein et al.^29^ validated this conclusion. As the index LV becomes more and more concerned, a crucial issue is the correct estimation of LV by mpMRI.^30^ In PI‐RADS v2.0, LV ≥0.5 ml was incorporate into the definition of csPCa.^15^ Recently, several studies have demonstrated the positive correlation between MRI LV and histological tumor volume, confirming that MRI slightly underestimates the true tumor volume within an acceptable range.^11^, ^13^, ^20^ With regard to the utility of LV in predicting malignant results, similar conclusion was reached in our study in accord with the studies above. In total, we found that 25.7% of patients with PI‐RADS 3 and LVs >0.5 ml had csPCa. As was shown in Supplementary S2, the detection rate increased with the increasing LV. With the lowest detection rate, 20 of 27 tumors were classified as Gleaso*n* = 3 + 3 in the LV ≤0.5 ml group. However, the detection rate was obviously increased in the 0.5–1 and ≥1 group, among which csPCa accounted for a significant proportion. Therefore, given the strong correlation between LV and tumor, subgroups of PI‐RAD 3 based on LV were included in our nomogram.

There were only two published studies focusing on nomograms of PI‐RADS 3 lesion. Zhang et al.^31^ established a model including PSAD, lesion region and ADC value (≤900 mm^2^/s). A threshold of 750–900 mm^2^/s was reported to assist in distinguishing between benign and malignant prostate tissues, below which ADC value was correlated with csPCa.^15^ In our study, however, ADC value ≤900 mm^2^/s was not an independent risk factor in the binary logistic regression while ADC value ≤750 mm^2^/s did. In addition, compared with the AUC of our model, varying degrees of decrease in AUC was found when applying data from the two institutions to constructing their nomogram. (Supplementary **S3.1** **and** **S3.2**). The reasons for this result may be different *b*‐values (high *b*‐value vs. moderate *b*‐value) and patient characteristics. Li et al.^31^ also developed a radiomics nomogram with an AUC of 0.840, which was quite different from us. In despite of the two nomograms above, they all ignored evaluation of patients with PSA <10 ng/ml in PI‐RADS 3 lesions, to whom urologists were harder to offer appropriate management recommendations.

It was well known that more PCa nodules were found in PZ in contrast to TZ,^32^ which is concordant with our study. However, there were also more benign and cisPCa lesions found in PZ. The difference between groups was insignificant both in the two institutions (*p* > 0.05). Thus, the location of PI‐RADS 3 lesions was excluded from our model. Plausible reasons for this phenomenon were as follows. Firstly, compared with PI‐RADS v2.0, atypical TZ nodules assigned Category 2 could be upgraded to Category 3 when showing markedly restricted diffusion (DWI‐upgraded TZ atypical nodules) in PI‐RADS v2.1, of which the PCa detection was similar to conventional TZ score three nodules.^4^, ^33^ This prominent change might weaken the weight of PZ in PCa detection. Furthermore, the confounding bias of different biopsy methods could account for the exclusion. Different from the study of Zhang et al.,^34^ the mixed biopsy approaches were used and the needle count difference in PZ and TZ was smaller in our study. In addition, the special characteristics of patients in our study (PI‐RADS 3, PSA <10 ng/ml) may also lead to the result.

Several limitations exist in the present study. First of all, the present study is a retrospective study with nature of selection bias. In the next place, subjective bias may exist for the reason that ROIs are segmented in a semi‐automatic way. Thirdly, the cohort of our study is based on the specially selected population (PI‐RADS 3, PSA <10 ng/ml) which could affect generalizability. However, this is exactly our advantage in predicting biopsy outcomes in the “double gray zone” patients. Finally, although PI‐RADS 3 subgroups are included in our model, large‐scale and well‐designed studies are warranted to validate the nomogram.

## 5 CONCLUSIONS

To our knowledge, this is the first study that applied PI‐RADS 3 subgroup into nomogram. Overall, the nomogram is a promising tool for predicting PCa and csPCa in biopsy‐naïve patients with PSA <10 ng/ml and PI‐RADS v2.1 = 3 lesions due to the good calibration and net benefit. Our model may assist urologists in biopsy decision making for the “double gray zone” patients.

## AUTHOR CONTRIBUTIONS

Can Hu and Jiale Sun performed the experiments, analyzed the data, contributed analysis tools and wrote the paper. Zhenyu Xu and Zhiyu Zhang performed the experiments, prepared figures and/or tables. Qi Zhou, Jiangnan Xu and Hao Chen analyzed the data, prepared figures and/or tables. Chao Wang and Jun Ouyang conceived and designed the experiments, reviewed drafts of the paper.

## FUNDING INFORMATION

This work was supported by Natural Science Foundation of Suzhou City (No. SLJ201906).

## CONFLICT OF INTEREST

The authors have no conflicts of interest to declare.

## ETHICS STATEMENT

The present study was conducted ethically in accordance with the provisions of the Declaration of Helsinki.

## Supporting information


Supplementary S1
Supplementary S2
Supplementary S3.1
Supplementary S3.2

## Data Availability

The authors confirm that the data supporting the findings of this study are available within the article and its supplementary materials. All the data were generated from institution 1 (FAHSU, The First Affiliated Hospital of Soochow University) and institution 2 (KS, Kunshan Chinese Medicine Hospital Affiliated to Nanjing University of Chinese Medicine).
