# Supplementary material for: Development and external validation of a novel nomogram to predict prostate cancer in biopsy‐naïve patients with PSA <10 ng/ml and PI‐RADS v2.1 = 3 lesions
Source: Cancer Med. 2022 Aug 3;12(3):2560–71. doi: 10.1002/cam4.5100 (PMC9939143; doi:10.1002/cam4.5100)
Supplement: Supplementary file 1 — Supplementary S1 Supplementary S2 Supplementary S3.1 Supplementary S3.2 [file CAM4-12-2560-s001.docx]

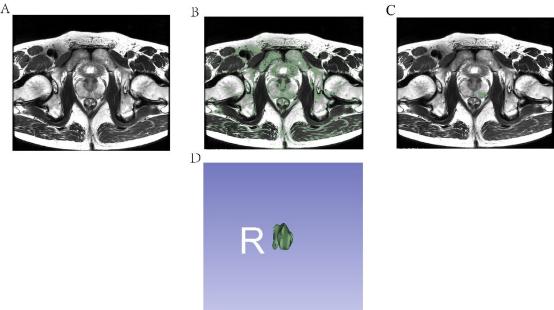


Supplementary 1: Semi-automatic three-dimensional segmentation of PI-RADS 3 lesions and the process of region of interest (ROI) drawing. Axial T2 weighted image (A); Threshold selecting (B); ROI segmentation (C); The 3D image (D).

Supplementary 2: The number of different Gleason scores according to LV subclassification of PI-RADS 3 lesions.

| LV, | Positive Gleason | | score, n |  |  | Upgraded, | Subtotal, | Benign |
| --- | --- | --- | --- | --- | --- | --- | --- | --- |
| ml | 3+3 | 3+4 | 4+3 | 4+4 | 4+5 | n (%) | n (%) |  |
| ≤ 0.5 | 20 | 3 | 4 | 0 | 0 | 3 ( 11. 1%) | 27( 13%) | 174 |
| 0.5- 1 | 12 | 11 | 5 | 4 | 2 | 8 (23.5%) | 34(44%) | 43 |
| ≥ 1 | 4 | 2 | 3 | 0 | 0 | 6 (66.7%) | 9(31%) | 20 |

Supplementary 3. 1: ROC curve comparing nomogram made by our study with made by

Zhang for PCa.

|  | Nomogram made by our study | | | | Nomogram made by Zhang’s study | | | |
| --- | --- | --- | --- | --- | --- | --- | --- | --- |
|  | AUC | SEN  (%) | SPE  (%) | P | AUC | SEN | SPE | P |
| Cohort 1 | 0.857 | 84.3 | 78.5 | <0.001 | 0.748 | 74.3 | 63.3 | <0.001 |
| External validation  group | 0.850 | 72.0 | 88. 1 | <0.001 | 0.821 | 72.0 | 81.0 | <0.001 |

Supplementary 3.2: ROC curve comparing nomogram made by our study with made by

Zhang for csPCa.

|  | Nomogram made by our study | | | | Nomogram made by Zhang’s study | | | |
| --- | --- | --- | --- | --- | --- | --- | --- | --- |
|  | AUC | SEN  (%) | SPE  (%) | P | AUC | SEN | SPE | P |
| Cohort 1 | 0.893 | 91.2 | 76.9 | <0.001 | 0.759 | 64.7 | 81.3 | <0.001 |
| External validation  group | 0.896 | 80.0 | 92.6 | <0.001 | 0.864 | 86.7 | 78.7 | <0.001 |
